# Health-related quality of life in children and adolescents allergic to staple foods

**DOI:** 10.1186/2045-7022-5-S3-O23

**Published:** 2015-03-30

**Authors:** Sven-Arne Jansson, Eva Östblom, Jennifer Protudjer, Marianne Heibert Arnlind, Ulf Bengtsson, Ingrid Källström-Bengtsson, Birgitta Marklund, Roelinde Middelveld, Georgios Rentzos, Ann-Christine Sundqvist, Johann Åkerström, Sven-Erik Dahlén, Staffan Ahlstedt

**Affiliations:** 1 The Centre for Allergy Research, Karolinska Institutet, Stockholm, Sweden; 2 Sachs' Children and Youth Hospital, Sodersjukhuset, Stockholm, Sweden; 3 Swedish Council on Health Technology Assessment, SBU, Stockholm, Sweden; 4 Allergy Unit, Sahlgrenska University Hospital, Gothenburg, Sweden; 5 The Swedish Asthma and Allergy Foundation, Stockholm, Sweden; 6 Department of Health and Caring Sciences, Linnaeus University, Kalmar, Sweden

## Background

Our group has previously described that food allergy negatively impacts health-related quality of life (HRQL) among adults. We now extend our investigation to children and adolescents.

## Objective

To investigate the factors that affect HRQL in households with a child or adolescent with objectively diagnosed allergy to at least one of the staple foods cow's milk, hen's egg or wheat. Further, to evaluate whether HRQL differs between cases and controls.

## Methods

Swedish children (0-12 years) and adolescents (13-17 years) with objectively diagnosed food allergy to staple foods were recruited in an outpatient allergy clinic. Age- and sex-matched controls were included. Food-allergic adolescents themselves and the parents of food-allergic children answered a food-allergy specific HRQL questionnaire (FAQLQ), developed within EuroPrevall. A generic HRQL questionnaire, EQ-5D, was answered by parents to cases and controls. In total, 85 children and 58 adolescents (cases), and 94 children and 56 adolescents (controls) participated.

## Results

We found that cases had lower HRQL measured by EQ-5D compared to controls (p<0.05). Using the disease-specific questionnaire, the domain most negatively affected by food allergy in adolescents was Allergen Avoidance and Dietary Restrictions, whereas Risk of Accidental Exposure had lowest impact. No significant differences were found between domain-specific scores for children. However, cardiovascular and respiratory symptoms significantly impaired HRQL in children. For adolescents, no particular symptom stood out. In children, previous anaphylaxis (p<0.001) and an epinephrine auto injector prescription (p<0.05) were associated with lower HRQL. This was not seen in adolescents.

## Conclusion

HRQL is poorer in children and adolescents with food allergy compared to controls. The use of a disease-specific questionnaire reveals that severity of disease is of importance. Some differences regarding effect on HRQL are seen between children and adolescents. This should be taken into consideration when managing pediatric patients of different ages with food allergies.

